# Surgical site infection among patients undergoing cardiac transplantation

**DOI:** 10.1186/2047-2994-4-S1-P75

**Published:** 2015-06-16

**Authors:** JASN Rodrigues, REL Ferretti-Rebustini, RNT Turrini, RA Lacerda, V de Brito Poveda

**Affiliations:** 1 Medical-Surgical, Nursing School of University of São Paulo, São Paulo, Brazil

## Introduction

The patient undergoing transplantation is susceptible to many diseases, especially the surgical site infections, which are the third leading cause of infection related to health care in Brazil.

## Objectives

To analyze the occurrence and the predisposing factors for surgical site infection in patients undergoing cardiac transplantation, verifying the relationship between infections and variables related to the patient and the surgical procedure.

## Methods

This retrospective cohort study was conducted exploring the medical records of patients older than 18 years who underwent heart transplantation. The correlation between variables was performed using the exact test of Fischer and Mann-Whitney-Wilcoxon.

## Results

The sample consisted of 86 patients, predominantly men, with severe systemic disease, underwent extensive preoperative hospitalization. Showed signs of surgical site infection 9.3% of transplant, five (62.5%) superficial incisional, two (25%) and deep (12.5%) of organ/space. There was no statistically significant association between variables related to the patient and the surgical procedure.

## Conclusion

There was no association between variables and cases of surgical site infection, possibly related to the small number of cases of infection in the sample investigated.

## Disclosure of interest

None declared.

